# Acute Exercise and Oxidative Stress: CrossFit^™^ vs. Treadmill Bout

**DOI:** 10.1515/hukin-2015-0064

**Published:** 2015-10-14

**Authors:** Brian Kliszczewicz, C. John Quindry, L. Daniel Blessing, D. Gretchen Oliver, R. Michael Esco, J. Kyle Taylor

**Affiliations:** 1 Kennesaw State University, Department of Exercise Science and Sports Management.; 2 Auburn University, Department of Kinesiology.; 3 University of Alabama, Department of Kinesiology.; 4 Auburn University at Montgomery, Department of Medical and Clinical Lab Sciences.

**Keywords:** antioxidant, high-intensity training, body-weight exercise

## Abstract

CrossFit^™^, a popular high-intensity training modality, has been the subject of scrutiny, with concerns of elevated risk of injury and health. Despite these concerns empirical evidence regarding physiologic stresses including acute oxidative stress is lacking. Therefore, the purpose of this investigation was to examine the acute redox response to a CrossFit^™^ bout. Furthermore, these findings were compared to a high-intensity treadmill bout as a point of reference. Ten males 26.4 ± 2.7 yrs having three or more months of CrossFit^™^ experience participated in the present study. Blood plasma was collected at four time points: Pre-exercise (PRE), immediately-post-exercise (IPE), 1 hr-post (1-HP) and 2 hr-post (2-HP), to examine oxidative damage and antioxidant capacity. Regarding plasma oxidative damage, CrossFit^™^ and Treadmill elicited a time-dependent increase of lipid peroxides 1-HP (CrossFit^™^=+143%, Treadmill=+115%) and 2-HP (CrossFit^™^=+256%, Treadmill+167%). Protein Carbonyls were increased IPE in CF only (+5%), while a time-dependent decrease occurred 1-HP (CrossFit^™^=−16%, Treadmill=−8%) and 2-HP (CF=−16%, TM=−1%) compared to IPE. Regarding antioxidant capacity, Ferric Reducing Antioxidant Power also demonstrated a time-dependent increase within CrossFit^™^ and Treadmill: IPE (CrossFit^™^=+25%, Treadmill=+17%), 1-HP (CrossFit^™^=+26%, Treadmill=+4.8%), 2-HP (CrossFit^™^=+20%, Treadmill=+12%). Total Enzymatic Antioxidant Capacity showed a time-dependent decrease in IPE (CrossFit^™^=−10%, Treadmill=−12%), 1-HP (CrossFit^™^=−12%, Treadmill=−6%), 2-HP (CrossFit^™^=−7%, Treadmill=−11%). No trial-dependent differences were observed in any biomarker of oxidative stress. The CrossFit^™^ bout elicited an acute blood oxidative stress response comparable to a traditional bout of high-intensity treadmill running. Results also confirm that exercise intensity and the time course of exercise recovery influence oxidative responses.

## Introduction

In recent years there has been a growing popularity in alternative styles of exercise training. According to the American College of Sports Medicine (ACSM) high-intensity interval training (HIIT) and body weight training are the top fitness trends of 2015 (Thompson, 2014). However, high-intensity training modalities have also been the subject of scrutiny, with concerns of elevated risk of injury and health (Bergeron et al., 2011). The chief program amongst these modalities is CrossFit^™^, a short duration (i.e. less than 30 minutes), high-volume, and high-intensity exercise program (Bergeron et al., 2011; Larsen and Jenson, 2014; Hak et al., 2013). Though similar to circuit training, CrossFit^™^ workouts typically do not provide structured periods of rest, allowing participants to be susceptible to elevated levels of exercise induced stress. Exercise creates alterations within the internal environment that can prove beneficial or harmful, depending on the magnitude of the stress stimulus (Powers and Jackson, 2008). One way to gauge internal environmental stress fluctuation is through biomarkers of oxidative stress.

Oxidative stress occurs when the oxidative damage through reactive oxygen species (ROS) surpasses endogenous antioxidant defense (Buettner, 1993). A prolonged increase of ROS is central to dyshomeostasis through the modification of protein and lipids and is influenced by exercise (Bloomer et al., 2005; Bloomer et al., 2007; Goldfarb et al., 2008; Miller et al., 2012; Nikolaidis et al., 2007). Moderate increases of ROS are beneficial, if not essential, for optimal physiological performance (Powers and Jackson, 2008), while excessive amounts hinder performance and recovery (Alessio and Hagerman, 2006; Powers and Jackson, 2008). Prolonged shifts of the internal environments favoring oxidation increases the likelihood of inflammation and promotion of cellular apoptosis (Fisher-Wellman and Bloomer, 2009). In regard to exercise, ROS production is proportional to the intensity or fatigue induced by duration of the bout, making it an adequate marker of exercise induced stress (Alessio et al., 2000; Alessio et al., 1988; Bloomer et al., 2007; Quindry et al., 2003).

Despite the growing popularity and concerns for potential risk, very little is known about the stress response to a CrossFit^™^ like bout of exercise. Therefore, the purpose of this study was to examine the blood oxidative stress response following a commonly performed bout of CrossFit^™^, “Cindy”. The workout “Cindy” (CF) is a 20 min bodyweight circuit, chosen because of its relatively light load and moderate time requirement, which are representative of common CrossFit^™^ exercise session duration and work volumes. To undertake this study, a comprehensive biomarker panel of blood oxidative damage and antioxidant defense measures was performed before and after the acute bout of CF. Furthermore, these findings were compared to a more traditionally prescribed high-intensity treadmill (TM) bout matched for time and approximate intensity as a point of reference.

## Material and Methods

### Participants

Ten apparently healthy males participated in this study. Each participant had a minimum of three months CF training experience. Participants were recruited through flyers and e-mails sent out to local CrossFit^™^ establishments. All participants were determined to be low risk for cardiovascular, metabolic, and/or pulmonary diseases as determined by the PAR-Q and Health History Questionnaire. Participants reported not taking any prescribed or over the counter medication during the time of the study. Subjects were instructed to abstain from exercise 24 hours prior to each trial, as well as caffeine, alcohol, and vitamin supplementation 12 hours prior.

### Measures

#### Oxidative Stress Biomarkers

Blood plasma samples were assayed for oxidative stress biomarkers as previously described (Hudson et al., 2008; Miller et al., 2012; Quindry et al., 2013). Two biomarkers were selected for oxidative damage, plasma lipid hydroperoxides (LOOH) and protein carbonyls (PC). Two additional biomarkers were selected to examine blood plasma antioxidant capacity, Ferric-reducing antioxidant power (FRAP) and Trolox-equivalent antioxidant capacity (TEAC).

Lipid peroxidation was determined through modified ferrous oxidation-xylenol orange assay (FOX) and reported as μmol/L (Nourooz-Zadeh et al., 1994). FOX measures the oxidation of ferrous ions to ferric ions by LOOH, which reacts with the ferrous-sensitive dye (xylenol orange). PC were assayed using a commercially available ELISA kit (Biocell, Papatoetoe New Zealand), protocols followed assay kit instructions and reported as μM/mg protein. Prior to analysis, individual plasma protein content was normalized using the methods of Bradford (1976). Antioxidant capacity was measured through the TEAC assay with modified methods from Re et al. (1999) and Villano et al. (2004). Though TEAC is often used to assess the antioxidant capacity of foods and beverages (Berg et al, 1999), it has also been used as a viable marker for antioxidant capacity in human serum (Cao and Prior, 1998). Total antioxidant potential was measured using the FRAP assay, which methods were adopted and modified from Benzie and Strain (1996). TEAC and FRAP are both assessed through colourimetric solutions spectrophotometrically (Benzie and Strain, 1996; Villaño et al., 2004). The biomarkers of oxidative stress selected for this study are sensitive to exercise application and have been used on several occasions by different labs (Bloomer et al., 2005; Hudson et al., 2008; Miller et al., 2012; Nikolaidis et al., 2007, 2008; Quindry et al., 2013). All biomarkers were normalized for plasma-volume changes that occurred following the exercise trials using the formulas based on the established protocols of Dill and Costill (1974).

### Procedures

#### Experimental Approach To The Problem

**Figure 1:**
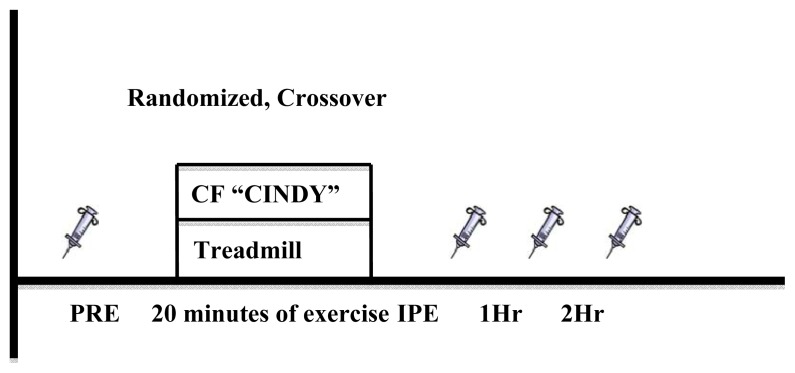
Study design. Biomarkers of oxidative stress (LOOH, PC, FRAP, TEAC) were taken at designated time points, represented by blood draw (syringe) icons. Samples taken before and after (randomized, crossover design) 20 minutes CF and TM bouts

The Institutional Review Boards of the Auburn University and Auburn University at Montgomery approved this study and protocols. Each participant arrived at the lab on three separate occasions. The first visit consisted of informed consent, health screening, familiarization of the protocols and a graded exercise test. The additional two visits included an exercise trial, between the times of 7–11 am. The exercise trials were carried out in a randomized-crossover fashion between the trials and observed by the primary investigator. Each visit was separated by 3–7 days. Participants were instructed to consume the same meal at the same time on the day of each visit. Venous blood was sampled before and after each trial by a trained phlebotomist. After initial collection participants performed a standardized 5 min warm up on the treadmill, rested for 1 min and then performed the exercise bout of CF or TM. Participants were not allowed to eat or drink during the 2-hour recovery period. The study design is presented in Figure 1.

#### Maximal Exercise Capacity and Anthropomorphic Measurements

Maximal exercise capacity (VO_2max_) and maximal heart rate (HR_max_) were assessed during the first session through a graded exercise test (GXT) on a treadmill (Trackmaster, Newton, KS). Using a Bruce Protocol, the workload during the GXT was increased incrementally every 3 min until a maximal value was reached. Expired gas (oxygen and carbon dioxide) fractions were sampled continuously using a pneumotach, mixing chamber, and gas analyzers through a Parvo Medics cart (Sandy, UT). During the GXT, the heart rate was assessed continuously using a heart rate monitor (Polar Electro Oy, Oulu, Finland). Test termination required achievement of two of the following criteria: a plateau in VO_2_ occurring with an increasing workload; the respiratory exchange ratio (RER) of > 1.10; the heart rate within 10 beats of age predicted maximum (220 – Age).

Body fat percentage was assessed through use of a total body Dual Energy X-Ray Absorptiometry (DXA) scan (GE Lunar Prodigy, Software version 10.50.086: GE Lunar, Corp., Madison, WI, USA). Subjects characteristics for aerobic and body composition data are presented in Table 1.

#### CrossFit^™^ Protocol

The CF workout “Cindy” protocol consisted of as many rounds possible of 5 pull-ups, 10 push-ups, and 15 air-squats in 20 min. Completion of all the prescribed repetitions for each movement is required before moving onto the next round. Each movement was held to CF standards for each participant and was enforced by the observing researcher. Upon completion participants were asked to assume a seated position for subsequent blood draws.

#### High-Intensity Treadmill Protocol

The TM bout required participants to run at a minimum intensity of the 90% maximal HR, which was determined upon the completion of the GXT. The target HR of 90% was determined via a pilot study, which examined the cardiovascular demand of “Cindy” (Kliszczewicz et al., 2014). Duration of the trial was timed-matched at 20-minutes. Participants were instructed to find a comfortable running speed, and then incline was continuously altered in order to yield the target HR. Upon completion participants were asked to assume a seated position for subsequent blood draws.

#### Blood Samples and Storage

Blood samples were collected and assayed for biomarkers of oxidative stress at the following time points: before exercise (PRE), immediately post exercise (IPE), 1-hour post exercise (1-HP), and 2-hours post exercise (2-HP). Participants were in a seated position while the 10mL blood samples were taken. Samples were taken via venipuncture through the antecubital vein and were collected in vacutainer EDTA tubes (2mL) and heparinized tubes (8mL). Heparinized tubes were inverted and immediately centrifuged at 3000 rpm for 15 minutes, with plasma aliquotted, and stored in ultra-low freezer −80°C until subsequent assay. Hematocrit and haemoglobin were determined using EDTA tube whole-blood aliquots using a hematology analyzer (CellDyn 1800, Abbott Park, IL).

### Statistical Analysis

A 2 (Trial) × 4 (Time) repeated measures analysis of variance (ANOVA) was used to assess differences from resting oxidative stress to post exercise values in and between both CrossFit (CF) and Treadmill trials (TM). For the key dependent variables, a Mauchly’s Test was run to determine whether there was no violation of sphericity. Statistical analysis was performed on SPSS 19.0 (Chicago, IL). A Tukey post hoc was used to determine significant mean differences when indicated. Statistical significance was set to α ≤0.05. Data are presented as means ± standard error of the mean (SEM).

## Results

All participants completed CF and TM protocols. Mean anthropomorphic values are presented in Table 1. In addition to monitoring the HR for intensity, a rate of perceived exertion (RPE) scale numbered 1–10, with 1 being no effort and 10 being maximal effort, was used. Percent HR_max_ and RPE were used to ensure a similar effort of intensity between trials; results are presented in Table 2.

**Figure 2:**
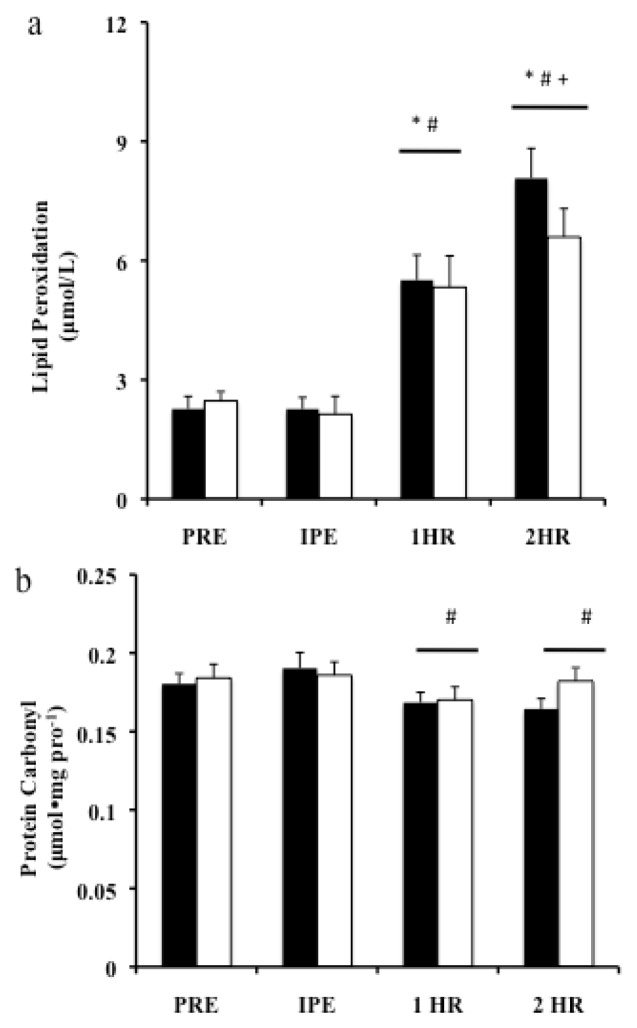
Biomarkers of Oxidative Damage, before and after exercise, Means ± SEM, n=10. a) lipid peroxidation of blood plasma b) Protein Carbonyl blood plasma. * significant from PRE, # significant from IPE, + significant from 1-HP. Black bars = CF, Open bars = TM.

Oxidative damage biomarkers are presented for blood plasma LOOH (Figure 2a) and blood plasma PC content (Figure 2b). Lipid damage (LOOH) demonstrated a significant time-dependent increase in both CF and TM trials (p<0.001), while no trial-dependent difference was observed (p=0.623). Plasma LOOH did not exhibit a significant increase until 1-HP (p<0.001) (CF= 170%± 36.9 and TM= 146%± 36.9). At 2-HP LOOH values increased further (CF= 351%± 107.3 and TM= 205%± 59.1) compared to PRE values (p< 0.001). A significant difference was observed between 1-HP and 2-HP (p=0.025). Analysis of mean plasma PC demonstrated a time-dependent decrease (p=0.001), but no trial-dependent differences (p=0.245). A non-significant increase was observed in IPE (p=0.187), (CF= 5.4%± 3.8 and TM= 1.8± 4.3), followed by significant decreases in both 1-HP (p=0.001) (CF= −10.6%± 2.7 and TM= −8.5%± 1.4) and 2-HP (p<0.001) (CF= 13.0%± 2.3 and TM= 3.5%± 10.2), only when compared to IPE.

**Figure 3:**
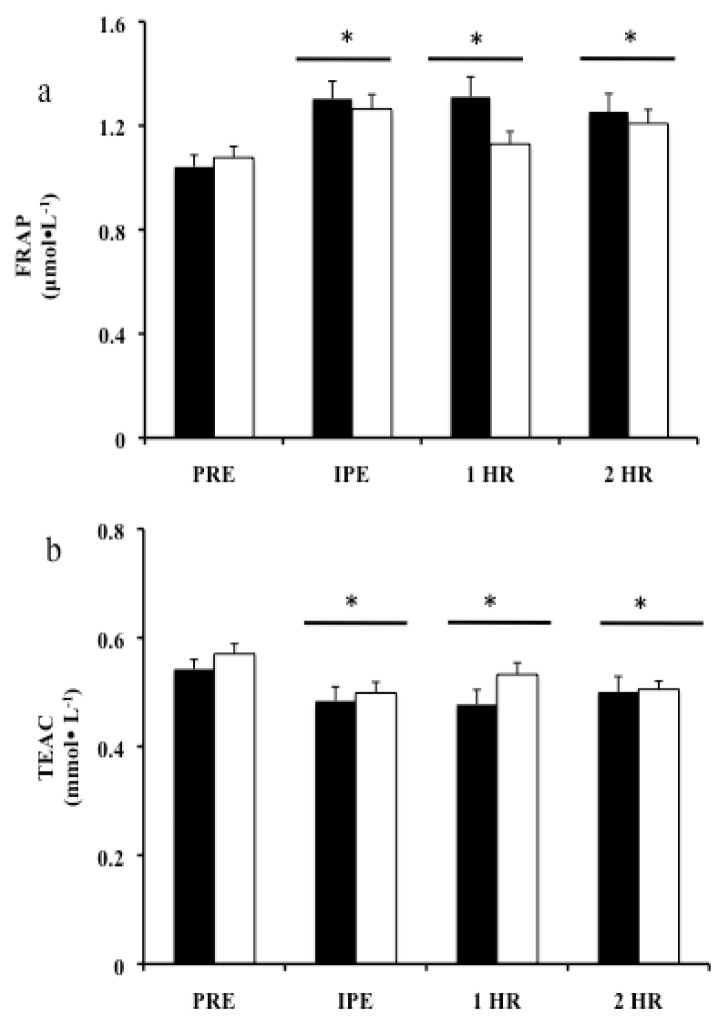
Biomarkers of Antioxidant Capacity. Means ± SEM, n=10. A) Blood plasma antioxidant potential FRAP B) Blood plasma antioxidant capacity TEAC. * Significantly different from PRE. Black bars = CF, Open bars = TM.

Biomarkers for antioxidant capacity are presented as FRAP (Figure 3a) and TEAC (Figure 3b). Analysis of FRAP demonstrated a significant time-dependent increase in both trials (p<0.001), but not between trials (p=0.080). FRAP concentration increased significantly in all post exercise time points IPE (p<0.001) (CF= 25.1%± 2.6 and TM= 17%± 2.2), 1-HP (p=0.001) (CF= 25.8%± 4.0 and TM= 4.9%± 2.6), and 2-HP (p=0.004) (CF= 20.8%± 5.1 and TM= 10.3%± 2.8). TEAC unexpectedly showed a negative time-dependent reaction in both trials (p<0.001), while no trial-dependent differences were observed (p=0.134). TEAC significantly decreased in all post measurements IPE (p<0.001) (CF= −11.28%± 2.4 and TM= −12.6%± 1.5), 1-HP (p=0.001) (CF= −12.5%± 2.6 and TM= −6.7%± 1.7), and 2-HP (p=0.001) (CF= −8.2%± 3.2 and TM= −9.3± 1.5).

## Discussion

The purpose of this investigation was to examine the acute blood oxidative stress response to a performed representative CrossFit^™^ exercise session. As a point of reference, findings from the CF trials were compared to TM bouts normalized for time and HR intensity. The key findings of this study were that the two bouts produced equivocal oxidative stress responses in terms of delineating mode-specific effects. These findings support results of previous studies suggesting that the oxidative stress response is more sensitive to the intensity of the exercise rather than the modality (Alessio et al., 2000; Quindry et al., 2003). In contrast to oxidative stress markers, the peak HR was statistically different, in that CF elicited a higher response than TM. However, HR responses between CF and TM are not believed currently to be of physiological significance, in that the average %HR_max_ in each trial is classified as vigorous intensity according to ACSM criteria (Garber et al., 2011). Similar to the HR, subjective quantification of exercise intensity by RPE also differed between trials, with CF eliciting a higher mean. Despite subtle differences in indices of exercise intensity, both CF and TM sessions were of high-intensity in terms of broad classifications. Collectively these findings suggest that the oxidative stress response is proportional to the exercise intensity performed. Additional points of consideration are provided below.

### Markers of Oxidative Stress

The marker for lipid damage, LOOH, increased in a trial-independent fashion following each bout and confirmed that exercise induced oxidative stress occurred. Previous works support these findings in that increasing measures of LOOH occur following several different exercise modalities such as hiking, cycling, and weight lifting (Hudson et al., 2008; Miller et al., 2012; Quindry et al., 2013). Quindry et al. (2003) observed that rises in LOOH occurred independently of aerobic metabolism, and significantly rose following exercise that surpassed the lactate threshold. Therefore, it is more likely that the exercise intensity of the bouts, approximately 90% of the HR_max_, was the driving force behind the lipid hydroperoxide production.

The time-dependent decrease in PC was an unexpected result of the study. PC is a sensitive marker of ROS mediated protein damage and often increases in plasma after bouts of acute exercise of varying intensities (Bloomer et al., 2007; Goldfarb et al., 2008; Hudson et al., 2008; Nikolaidis et al., 2008; Quindry et al., 2011, 2013). However, the current observation is not the first of such instances where exercise does not result in an increase in PC (Bloomer et al., 2006). Bloomer et al. (2006) observed no significant change in PC following an acute bout of squats or sprints, although interestingly a compelling decrease in the numeric response was noted. Similarly, Quindry et al. (2011) observed no change immediately post eccentric exercise; however, 24-hours following the session, a significant rise in PC was observed. This suggests that the window of time in this study may not have been sufficient enough to observe a response.

### Markers of Antioxidant Defense

The antioxidant potential, ascorbate equivalent (FRAP), measures the antioxidant reducing ability in plasma and is commonly elevated in response to rising levels of ROS (Benzie and Strain, 1996). Plasma levels of FRAP increased significantly IPE and remained elevated throughout the time points following CF and TM protocols, an expected response due to the presence of oxidative stress in both trials. Elevation of plasma FRAP is well documented in prior investigations that employed various exercise modalities ranging from aerobic to anaerobic (Quindry et al., 2013). Given that FRAP is most sensitive to changes in uric acid, this finding reflects accelerated purine metabolism during the exercise recovery (Sutton et al., 1980; Cao and Prior, 1998). Moreover, elevations in plasma FRAP/uric acid only occur following high-intensity muscular activity and further reinforce the notion that CF and TM bouts elicited similar physiologic stress responses.

Antioxidant capacity measured through TEAC decreased significantly following both trials. A decrease in TEAC is unusual in the presence of oxidative damage coupled with increased antioxidant potential. However, prior studies have observed a decrease in TEAC following exercise (Falone et al., 2010; Hudson et al., 2008). The cause of the observed decreases in TEAC cannot be completely resolved currently.

### Limitations

Although this study was a novel step towards the better understanding of oxidative stress following a bout such as CF, it was not without limitations. The marker used to control for intensity (%HR_max_) was similar between trials, but statistically different. The observed marker of intensity (RPE) also significantly differed between trials; however, psychological perception and influences may be responsible for the observed differences. In light of these findings, future studies should apply a more accurate marker to control for exercise intensity such as %VO_2max_. In conjunction to this, post exercise measures of lactate should be taken to examine the anaerobic component out the bout, as well as to assure matching intensity.

Future research should include creatine kinase (CK) and lactate dehydrogenase (LDH) to the biomarker panel. These biomarkers are released during periods of cellular damage and can provide telling information when compared to oxidative stress biomarkers (Banfi et al., 2012). Various styles of CrossFit^™^ workouts involving complex Olympic/gymnastic movements, heavier loads and shorter duration should also be examined in order to provide a more holistic representation. In addition, the participants of this study were trained and experienced with high-intensity exercise; untrained participants should be examined in order to gain a better understanding of the physiological stresses involved in acute CrossFit^™^ bouts. Furthermore, since the redox time course was not evaluated beyond two-hours into recovery, future studies should examine high-intensity bouts with a more extensive timeline.

## Conclusion

The observed oxidative stress response to the representative CrossFit^™^ workout “Cindy” elicited several telltale markers of blood oxidative stress in a time-dependent fashion. Just as importantly, these responses were statistically identical to a more commonly prescribed and less scrutinized bout of high-intensity treadmill exercise. Based on these findings, we interpret the oxidative stress outcomes of the current study to indicate that when closely matched for exercise time and intensity, the CrossFit^™^ bout “Cindy” produces a similar physiological stress response to a running modality.

## Figures and Tables

**Table 1:** Participants’ Characteristics

Characteristic	Values ± SEM
Age (yr)	26.4 ± 0.9
Body Height (cm)	177.3 ± 2.4
Body Mass (kg)	82.6 ± 1.2
Body Fat (%)	11.2 ± 2.4
VO_2max_ (ml·kg^−1^·min^−1^)	44.4 ± 5.1
Max HR (beats·min−1)	183.6 ± 2.0

**Table 2:** Exercise Intensity (Perceived and %HRmax)

	6-min	10-min	16-min	20-min	p
**RPE**					
**CF**	5.4 ± 0.5	6.4 ± 0.5	7.6 ± 0.2	9.0 ± 0.3	p= 0.005 (time)
**TM**	4.6 ± 0.4	5.6 ± 0.4	7.2 ± 0.4	7.2 ± 0.5	p= 0.007 (trial)
**%HRmax (**beats·min−1**)**					
**CF**	93.3 ± 1.2	94.6 ± 0.9	95.9 ± 1.0	97.7 ± 1.9	p= 0.005 (time)
**TM**	89.3 ± 1.1	92.9 ± 0.8	94.2 ± 0.9	93.6 ± 1.0	p< 0.001 (trial)

**N=10 Means +/− SEM.** RPE scale 1–10 lowest to highest exertion.
